# Deadly Nightshade, or Belladonna1Read before the meeting of the Genesee Valley Veterinary Medical Association, January 12. 1899.

**Published:** 1899-08

**Authors:** G. C. Kesler

**Affiliations:** Holly, N. Y.


### DEADLY NIGHTSHADE, OR BELLADONNA.[1]

By G. C. Kesler, V.S.,
HOLLY, N. Y.

Deadly nightshade, or belladonna, of which you all know, grows wild in most parts of Great Britain, especially around old walls, hedges of plantations, ruins, and shady places, and is now cultivated at Hitchin and elsewhere. It also grows wild in this country. I have seen it in Orleans County, N. Y., where I have had the sad experience of meeting with it in all of its characteristic forms. Here it varies somewhat in its habitat; we find it in fence-corners, around stone-piles, walls, stumps, marshy and shady places.

The appearance of this bush or vine is well described in the materia medica: dark-green vine or bush, resembling a tomato-vine, except in June or July, when it has pendulous, dark-purple or blue, bell-shaped flowers; in August and September it has round, violet-berried fruit the size of small cherries, which hang in a bunch, and have kidney-shaped seeds with a mild odor. When once seen it is easy to remember.

Cattle, sheep, and goats are the victims of this plant, because it is natural for them to browse and leave good feed. I came in contact with one pasture this last July and found four head of cattle dead and two sick. I diagnosed the case, and found the weed where they had browsed the leaves off, and convinced the owner. He said that last summer he had lost twelve sheep; in fact every year he had lost some in the same pasture. I cite this case as one of a great many.

*Diagnostic symptoms.* The eyes glassy; pupil dilated; secretions suspended; rumination ceased; insists on lying down; when compelled to rise, if possible, staggers behind; dull or drowsy, no desire for the rest of the stock; sometimes one or more quarters of udder swells. Later symptoms: discharge from eyes and nose, slight cough, and snoring or whistling sound on slight exertion.

*Result.* Recover, dry up, or die.

*Treatment.* Depends on the time you see them after they have eat the poison. Antidote: Stimulants of the bowels, kidneys, and skin.

[1] Read before the meeting of the Genesee Valley Veterinary Medical Association, January 12. 1899.

I believe that, sometimes, cases are diagnosed and treated as something else by veterinarians, two instances of which I know. Owing to a lack of literature touching on this condition, we are apt to be careless and not spend sufficient time to study it out. I bring this subject to your attention to encourage a closer investigation, and more complete record of such obscure cases, that we may be better fitted to advise with our clients in the removal of the offending cause.

